# Evaluation of the Bluetooth-enabled Scanbo device for point-of-care measurement of blood pressure and blood glucose: A cross-sectional pilot study in an urban slum of Bengaluru

**DOI:** 10.1016/j.fhj.2025.100271

**Published:** 2025-08-06

**Authors:** Venkatesh Karthikeyan, Sathyanarayana Tamysetty Narayan, Chinnu Sara Varughese, Shamshad Ahmad, Haripriya H, Mohit Bhardwaj

**Affiliations:** a Department of Community and Family Medicine, All India Institute of Medical Sciences Patna, Bihar, India; b Indian Institute of Public Health, Bengaluru, Karnataka, India; c Department of Community Medicine, Pushpagiri Institute of Medical Sciences and Research Centre, Thiruvalla, Kerala, India

**Keywords:** Diabetes, NCD, Hypertension, Point-of-care devices, Point-of-care technology, Non-communicable diseases

## Abstract

**Background:**

As non-communicable diseases escalate globally, timely and accurate monitoring of health indicators like blood pressure and blood glucose becomes critical. The Scanbo device, a Bluetooth-enabled point-of-care tool, promises to revolutionise this monitoring by offering quick and reliable measurements.

**Objectives:**

To validate the concordance of the results of the Scanbo device compared with the Omron for blood pressure and with the diagnostic lab method for blood sugar.

**Methods:**

This observational cross-sectional pilot study was conducted in the urban slum of Vijinapura (Bengaluru) to evaluate the accuracy of the Scanbo device. We enrolled 128 participants for blood pressure assessments and 69 for blood glucose measurement, selecting adults from the community based on predefined criteria. The primary variables measured included systolic and diastolic blood pressures and blood glucose levels, with data collected using both the Scanbo device and standard measurement methods for comparison. Data were recorded and analysed to ensure consistency and reliability. Statistical analyses were performed using Jamovi 2.3.28 solid version (The Jamovi project, 2021, https://www.jamovi.org), employing Bland–Altman plots to assess agreement, Cohen’s kappa for inter-rater reliability, and ROC curves to determine diagnostic accuracy. Sensitivity, specificity, positive predictive value and negative predictive value were also estimated.

**Results:**

The Scanbo device showed excellent accuracy in measuring systolic blood pressure (AUC = 0.985) and blood glucose (AUC = 0.973), with substantial agreement in reliability (Cohen’s kappa > 0.67 for all measurements). Diastolic blood pressure measurements were also accurate (AUC = 0.924), although slightly less so than systolic measurements. The mean differences between the Scanbo and standard measurements, sensitivity, specificity, positive predictive value and negative predictive value were within clinically acceptable limits.

**Conclusions:**

The Scanbo device demonstrates a high level of accuracy and reliability comparable to standard methods for systolic and diastolic blood pressure and blood glucose measurements. These findings support the potential integration of the Scanbo device into routine clinical practice, particularly for community health settings and patient self-monitoring.

## Highlights

• The Bluetooth-enabled Scanbo device demonstrated high accuracy in measuring blood pressure and blood glucose in a resource-limited urban setting.
• Significant concordance was observed between Scanbo and standard methods, especially for systolic blood pressure (AUC: 0.985) and blood glucose (AUC: 0.973).
• The device’s ease of use and reliable data transfer make it a promising tool for point-of-care diagnostics, particularly in underserved areas.
• Findings support the potential of integrating the Scanbo device into routine care for effective monitoring and management of non-communicable diseases.

## Introduction

The Global Burden of Disease (GBD) categorises diseases into three main groups: communicable (infectious diseases along with maternal, perinatal and nutritional conditions), non-communicable diseases (NCDs) and injuries.^1^ NCDs account for 74% of all mortalities worldwide, with the World Health Organization (WHO) reporting approximately 41 million NCD-related deaths annually.^2^ Cardiovascular diseases are notably significant, resulting in 17.9 million deaths each year, while diabetes contributes to 2 million fatalities annually.^2^ The impact of NCDs is disproportionately higher in low- and middle-income countries, which experience 77% of the global deaths attributed to NCDs.^2^

In India NCDs have become a major public health concern, with an estimated 5.8 million deaths attributable to NCDs annually.^3^ Approximately one in four Indians is at risk of premature death from an NCD before the age of 70.^4^ The country is undergoing a rapid epidemiological transition, with the burden of disease shifting from communicable diseases to NCDs.^5^ The mortality and morbidity due to NCDs have surged dramatically, rising from 37.9% in 1990 to 61.8% in 2016.^6^ NCDs also contribute significantly to poor quality of life, as indicated by the increase in disability-adjusted life years (DALYs) from 30% in 1990 to 55% in 2016.^3^ According to the National Family Health Survey-5 (NFHS-5), the prevalence of hypertension in the 45–49 age group is 31.6% among women and 34.1% among men.^7^ Additionally, 8.9% of women and 14.4% of men in the same age group have random blood glucose levels above 160 mg/dL.^7^ Studies show that NCDs account for 40% of all hospital stays and around 38% of all outpatient visits in India.^4^ In Karnataka, the NCD burden accounts for around 25% of the total disease burden among individuals aged 15–39 years, and for those over 40 years of age, NCDs constitute more than 70% of the total disease burden.^8^

As India progresses through a demographic and epidemiological transition, the composition of its population is shifting towards a higher proportion of middle-aged and older individuals.^9^ This shift is expected to substantially increase the burden of NCDs in the coming decades, and underscores the urgency of implementing effective and scalable healthcare interventions. Point-of-care testing (POCT) represents a pivotal advancement in this regard. Point-of-care diagnostics enables rapid diagnostics directly at the site of patient care, facilitating timely decision-making and potentially reducing the need for multiple patient visits.^10^ This is particularly advantageous in regions where access to comprehensive laboratory services is limited. By integrating technologies like POCT into primary healthcare systems, we can significantly enhance the management of chronic conditions such as hypertension and diabetes, which require continuous monitoring.

The rationale behind this study is to assess the effectiveness of a novel Bluetooth-enabled POCT device, Scanbo, which promises to streamline the monitoring process of blood pressure and blood glucose levels. These devices are crucial for patient management in primary healthcare settings, offering the advantages of rapid testing, immediate results, error-free data and data security, which are vital for chronic disease management in dynamically changing health statuses.^11^ The novelty of this study lies in the validation of the Scanbo device against conventional diagnostic methods. By comparing its accuracy and reliability, this research aims to contribute valuable insights into the feasibility of wider adoption of such technologies in India’s healthcare landscape, particularly in underserved areas. This could potentially transform the approach to NCD management by making diagnostic processes and record-keeping more efficient and patient-friendly, thereby improving overall health outcomes and compliance. Thus, this study is carried out with the objective of validating the concordance of the results of the Scanbo device (Bluetooth enabled) compared with the Omron for blood pressure and with the diagnostic lab method for blood sugar.

## Materials and methods

### Study design

This observational cross-sectional study was conducted to assess the concordance between measurements obtained from the Bluetooth-enabled Scanbo device and traditional methods, using the Omron device for blood pressure and a standard laboratory method for blood sugar.

### Study setting

The study was carried out in the urban slum of Vijinapura, a public health field site affiliated with the Indian Institute of Public Health, Bengaluru, India, from January to May 2021. During this time, the study team interacted with community leaders and local healthcare providers to facilitate participant enrolment and ensure the smooth execution of the study procedures.

### Study participants

Participants were eligible for inclusion if they were aged 30 years and above, residents of the study area, and willing to participate in the study after obtaining written informed consent. Exclusion criteria included individuals with conditions affecting blood pressure and blood sugar measurements, such as atrial fibrillation or severe peripheral arterial diseases, and those unwilling to provide consent. Convenience sampling was used to recruit the study participants.

### Variables

The primary variables measured were systolic and diastolic blood pressure (in mmHg) and blood glucose levels (in mg/dL). Measurements were obtained using both Scanbo (Scanbo India Private Limited, Sudama Chowk Road, Surat, Gujarat, India)^12^ and Omron (Omron Healthcare India Private Limited, Sector – 18, Udyog Vihar, Gurgaon, Haryana)^13^ devices for blood pressure. The Scanbo device and a standard laboratory glucose assay were used for the measurement of blood sugar. To reduce potential bias in device measurements, each device was calibrated according to the manufacturer’s specifications before the start of the study.

Individuals were classified as hypertensive if their systolic blood pressure exceeded 140 mmHg or their diastolic blood pressure exceeded 90 mmHg, as measured by the Omron device. Similarly, individuals were identified as having high blood glucose if their blood glucose levels surpassed 140 mg/dL, as determined by a standard laboratory glucose assay. Concordance between the devices was the main outcome measure, assessed through various statistical metrics including Bland-Altman plots, Cohen’s kappa, area under the curve, sensitivity, specificity, positive predictive value and negative predictive value.

### Measurement

Scanbo is a Bluetooth-enabled point-of-care diagnostic device.^2^ The Scanbo device measures the blood pressure of the upper arm and has a pressure range of 0–299 mmHg. It also measures blood sugar using the dry strip method. There is an automatic data transfer mechanism from the device to the mobile app and the data match the phone number of beneficiaries.

Blood pressure was measured under controlled conditions – participants were seated comfortably with their back supported, legs uncrossed, and arm positioned at heart level. Each participant rested for at least 5 minutes before measurements were taken. The Omron device was used first, followed by the Scanbo device. To minimise potential biases from the order of device use, a 1-minute wait period was maintained between the use of the two devices. This sequence was consistently adhered to throughout the study for all participants. Three readings were taken alternately from each device, and the average of the last two readings from each device was used for analysis. A mean difference of ≤5 mmHg with SD ≤8 mmHg for systolic and diastolic BP is considered tolerable.^14^

**Fig. 1:**
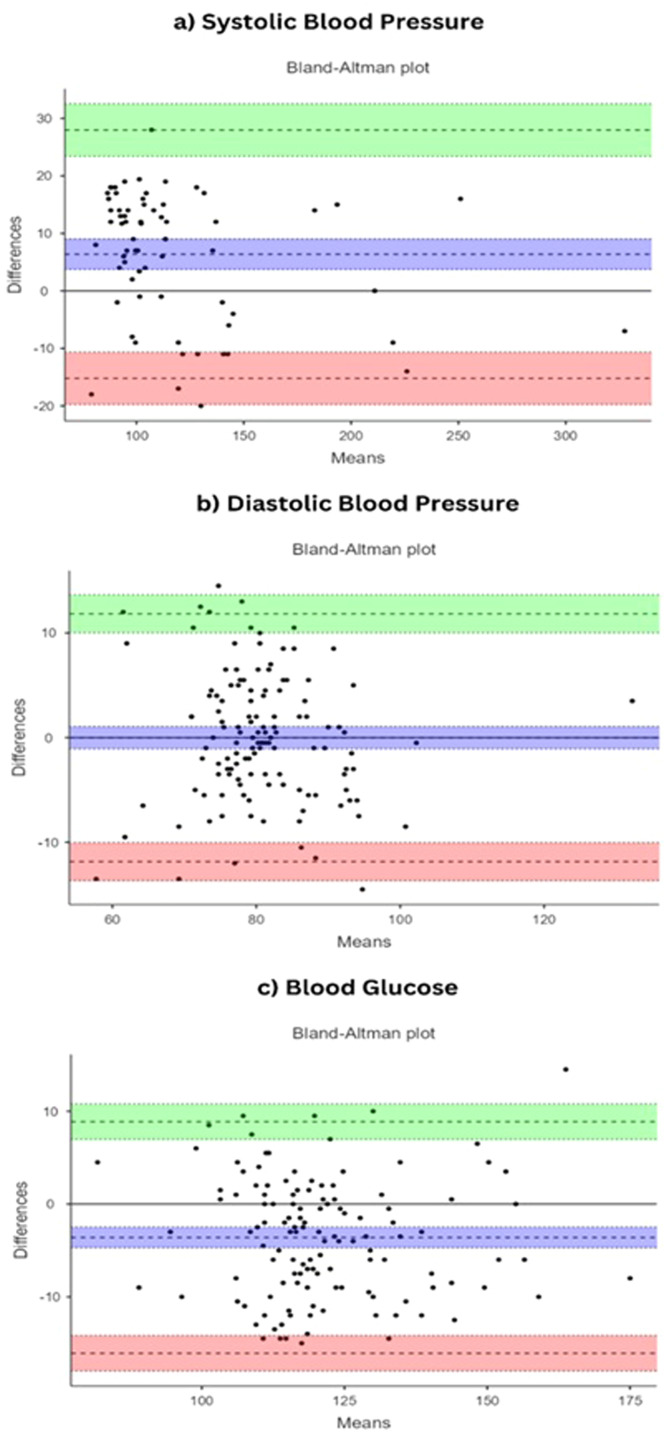
Bland–Altman plots illustrating the agreement between standard and Scanbo device measurements for systolic blood pressure, diastolic blood pressure and blood glucose.

Blood glucose measurements were taken after an overnight fast. After proper sterilisation, 2 mL of venous blood was obtained from the forearm of the participant and placed in fluoride oxalate tubes. The blood sample was sent to the laboratory within 2 hours. The Scanbo device for glucose check used a dry chemistry strip, ie electrochemical method involving a variant of the enzyme glucose dehydrogenase that was impregnated into a strip. A drop of blood was taken from the fingertip and placed on the Scanbo device glucose slot, and the reading displayed on the mobile Scanbo app was documented. Each set of readings was documented by the same trained personnel and the laboratory assay was done by a trained, experienced laboratory technician (Fig. 1). A difference of ≤15 mg/dL between both devices is considered tolerable.^15^

### Study size

A total of 128 study participants for blood pressure measurement and 69 participants for blood glucose estimation were enrolled in this pilot study, considering the logistical and resource constraints. This sample size was intended to provide initial insights into the performance of the Scanbo device in a real-world setting, with the expectation that findings would guide further research and possibly larger subsequent studies.

### Statistical analysis

Data analysis was performed using Jamovi 2.3.28 solid version (The Jamovi project, 2021, https://www.jamovi.org). Descriptive statistics included means and standard deviations to summarise participant characteristics and main study variables. To assess the agreement between the Scanbo and Omron devices for blood pressure, and between the Scanbo device and standard laboratory assays for glucose, Bland–Altman plots were used to analyse mean differences and limits of agreement. For the diagnostic performance of the devices, receiver operating characteristic (ROC) curves were generated, and the area under the curve (AUC) was calculated to assess the overall accuracy. Sensitivity and specificity were determined to evaluate the ability of the Scanbo device to correctly identify hypertensive and hyperglycaemic conditions compared to the standard methods. Positive predictive value (PPV) and negative predictive value (NPV) were also calculated to provide insights into the clinical utility of the Scanbo device in detecting these conditions. Cohen’s kappa was employed to measure inter-rater agreement on categorical classifications, with values interpreted as follows: 0.61–0.80 indicating substantial agreement and values above 0.80 indicating almost perfect agreement. All tests were two-tailed, and a p-value of less than 0.05 was considered statistically significant.

## Results

In the present study, blood pressure measurements were obtained from a total of 128 participants, comprising 70 men (54.7%) and 58 women (45.3%). The overall mean age of the individuals assessed for blood pressure was 42.1 years, with a standard deviation of 14.4 years, indicating a diverse age distribution among the subjects. For blood glucose measurements, the study included 69 participants, of whom 41 were male (59.4%) and 28 were female (40.6%). The participants in the blood glucose measurement group had a higher mean age of 48.2 years, with a standard deviation of 9.4 years, reflecting a slightly older subset compared to the blood pressure group.

In the study, the descriptive statistics for blood pressure and blood glucose indicate a normal distribution for both sets of data, as confirmed by visual inspection of Q-Q plots. As shown in Table 1, findings revealed that the mean systolic blood pressure (SBP) measured by the standard method was 123 mmHg (SD = 15.2), while the Scanbo device recorded a mean SBP of 120 mmHg (SD = 14.9). The mean difference in SBP between the two methods was 3.6 mmHg (SD = 6.37), with statistical analysis showing a significant difference (p < 0.001). However, this difference is clinically acceptable. Diastolic blood pressure (DBP) showed a mean of 80.9 mmHg (SD = 9.59) for both methods, with a negligible mean difference of 0.00781 mmHg (SD = 6.04), indicating no significant difference statistically (p = 0.909) and clinically. For blood glucose measurements, the standard method showed a mean of 116 mg/dL (SD = 45.4), compared to 122 mg/dL (SD = 42.2) measured by the Scanbo device, with a mean difference of -6.38 mg/dl (SD = 11.0) that was statistically significant (p < 0.001) but clinically acceptable. Among the participants, 20 (15.6%) were identified with an SBP exceeding 140 mmHg, and 23 (18%) participants had a DBP over 90 mmHg, as measured by the Omron device. Furthermore, 13 participants, representing 18.8% of the sample, recorded blood glucose levels above 140 mg/dL, as determined by standard laboratory glucose assays. These findings provide a quantitative basis for evaluating the performance of the diagnostic devices used in the study.

**Table 1:** Comparison of blood pressure (*N* = 128) and blood glucose (*N* = 69) measurements between standard methods and the scanbo device.

Variable	Mean (±SD) by standard method	Mean (±SD) by Scanbo device	Mean difference (±SD) between Scanbo and standard method	p-value (paired samples t-test)
Systolic blood pressure (in mmHg)	123 (±15.2)	120 (±14.9)	3.6 (±6.37)	<0.001
Diastolic blood pressure (in mmHg)	80.9(±9.59)	80.9 (±9.15)	0.00781 (±6.04)	0.909
Blood glucose (in mg/dL)	116 (±45.4)	122 (±42.2)	−6.38 (±11.0)	<0.001

The Bland–Altman plots presented in Fig. 1 offer a visual assessment of the agreement between the measurements obtained using the standard method and those acquired with the Scanbo device across three different health metrics: systolic blood pressure, diastolic blood pressure and blood glucose levels. The plot for systolic blood pressure indicates a mean difference of 3.6 mmHg, with most data points lying within the limits of agreement, which span from -8.88 to 16.08 mmHg. The diastolic blood pressure plot shows a very tight mean difference of 0.00781 mmHg, with limits of agreement ranging from -11.83 to 11.84 mmHg. The clustering of data points around the zero difference line indicates a high level of agreement between the two measurement methods. For blood glucose, the mean difference is -6.38 mg/dL, with limits of agreement extending from -27.97 to 15.22 mg/dL. The distribution of differences shows that the Scanbo device tends to record higher glucose values than the laboratory method. Overall, the Bland–Altman plots demonstrate good agreement for diastolic blood pressure measurements between the Scanbo device and the standard method, and acceptable agreement for systolic blood pressure and blood glucose measurements.

The analysis of the receiver operating characteristic (ROC) curves demonstrates high diagnostic accuracy of the Scanbo device across all measured parameters. The area under the curve (AUC) for systolic blood pressure (SBP) is exceptionally high at 0.985 (0.968 1.000), indicating excellent diagnostic ability to distinguish between normotensive and hypertensive individuals. For diastolic blood pressure (DBP), the AUC is 0.924 (0.873 0.975), which also reflects very good accuracy, but suggests a slightly lower discriminative power compared to SBP. The blood glucose measurements show an AUC of 0.973 (0.940 1.000), nearly matching the high accuracy observed in SBP measurements, and indicating that the Scanbo device is highly effective in identifying normal and elevated blood glucose levels.

**Fig. 2:**
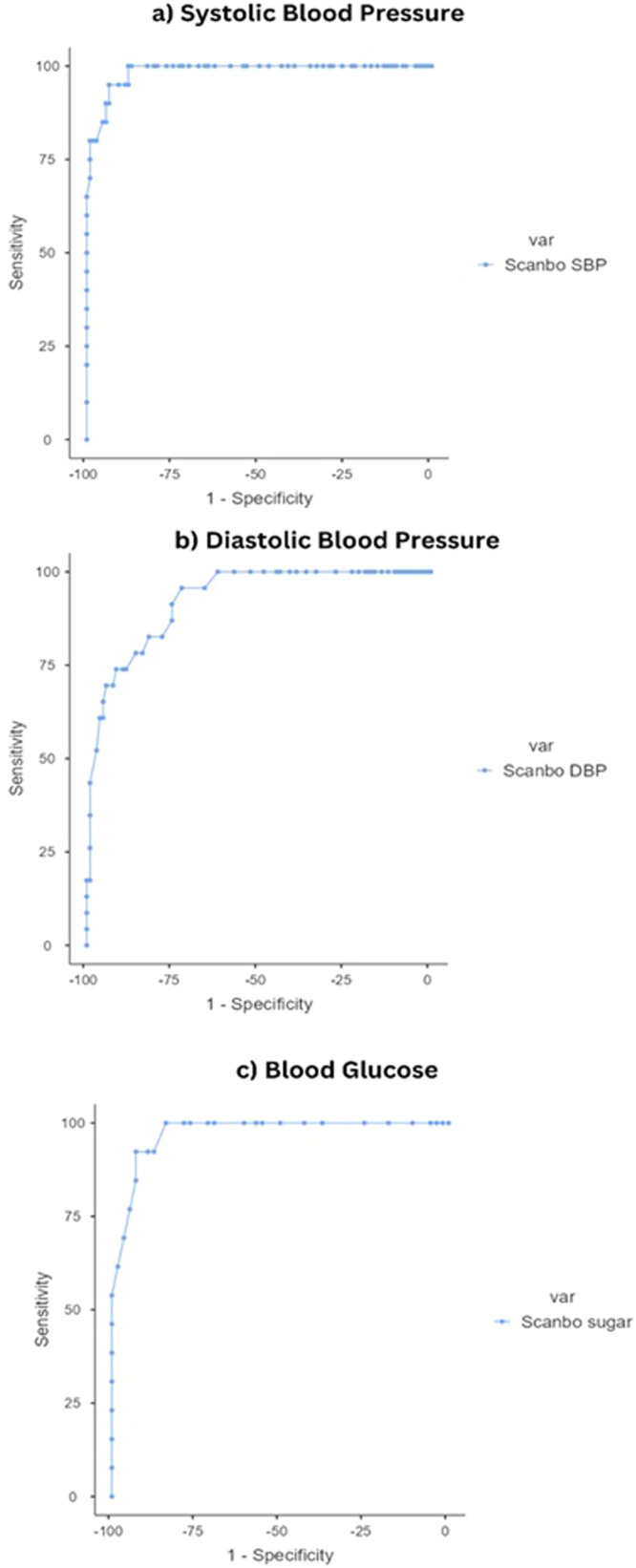
Receiver operating characteristic (ROC) curves for the diagnostic accuracy of the Scanbo device in measuring systolic blood pressure, diastolic blood pressure, and blood glucose

The ROC curves presented in Fig. 2 affirm that the Scanbo device performs robustly in clinical assessments, with AUC values approaching 1.0, which is considered outstanding in diagnostic tests. The steep ascent in the curves across all three parameters – particularly noticeable in the initial thresholds – reinforces the device’s sensitivity and specificity, making it a reliable tool for POCT in diverse settings. This efficacy is crucial for timely and accurate health monitoring, significantly impacting clinical decision-making and patient management for hypertension and diabetes.

The statistical analysis presented in Table 2 provides a detailed evaluation of the performance of the Scanbo device in measuring systolic and diastolic blood pressure, as well as blood glucose levels, using specific cutoffs for each parameter.

**Table 2:** Statistical analysis of measurement accuracy for systolic blood pressure, diastolic blood pressure, and blood glucose using the Scanbo device.

Parameters	Systolic blood pressure	Diastolic blood pressure	Blood glucose
Cohen’s kappa	0.673	0.624	0.698
Sensitivity	94.4%	94.29%	92.86%
Specificity	90%	69.57%	92.31%
PPV	98.08%	93.4%	98.11%
NPV	75%	72.73%	75%

Systolic blood pressure (SBP): The analysis utilises a cutoff of 129 mmHg for SBP. The diagnostic performance is excellent, with high sensitivity (94.4%) and good specificity (90%). The positive predictive value (PPV) is 98.08%, and the negative predictive value (NPV) is 75%, indicating reliable diagnostic capability at this cutoff. The Cohen’s kappa of 0.673 reflects substantial agreement.

Diastolic blood pressure (DBP): For DBP, a cutoff of 88 mmHg was used. Despite a somewhat lower specificity at 69.57%, the sensitivity remains high at 94.29%, with a PPV of 93.4% and an NPV of 72.73%. The Cohen’s kappa of 0.624 suggests very good accuracy.

Blood glucose: A cutoff of 123 mg/dL for blood glucose was used for analysis. Both sensitivity and specificity are robust at 92.86% and 92.31%, respectively, supported by a very high PPV of 98.11% and an NPV of 75%. The Cohen’s kappa of 0.698 demonstrates excellent diagnostic accuracy.

## Discussion

The present study aimed to assess the accuracy and reliability of the Scanbo device for measuring systolic blood pressure, diastolic blood pressure and blood glucose levels compared to standard measurement methods. The findings reveal that the Scanbo device consistently provides high diagnostic accuracy for systolic blood pressure, with an area under the curve (AUC) of 0.985, demonstrating exceptional ability to discriminate between hypertensive and normotensive states. Similarly, the device shows excellent accuracy for blood glucose measurements with an AUC of 0.973. However, while still displaying good diagnostic performance, the diastolic blood pressure measurements had a slightly lower AUC of 0.924, indicating a small reduction in accuracy compared to systolic measurements. The substantial agreement in measurement comparisons, as evidenced by Cohen’s kappa values ranging from 0.624 to 0.698, confirms the device’s reliability across the three tested parameters.

The findings of this study are largely consistent with those reported in similar research evaluating the accuracy of point-of-care diagnostic devices. The high diagnostic accuracy for systolic blood pressure (AUC = 0.985) in our study aligns with findings from other research, such as the study by Wetterholm *et al*.,^6^ which reported a Spearman correlation coefficient of r=0.78 for SBP using the Andersson Lifesense BDR 2.0 and Beurer BM 85 devices, underscoring strong concordance with standard clinical measurements (p<0.001). Similarly, the good performance of the Scanbo device in measuring blood glucose (AUC = 0.973) is in agreement with Bailey *et al*.,^16^ who reported comparable accuracy for a wireless blood glucose monitoring system, highlighting the reliability of such devices in routine clinical use. However, the slightly lower AUC for diastolic blood pressure (0.924) observed in this study contrasts with findings from Takahashi *et al*.,^17^ who reported higher concordance for diastolic readings in their evaluation of multiple automated blood pressure devices. This discrepancy may be attributed to differences in device technology, study population or measurement protocols, suggesting a need for further investigation into factors influencing diastolic pressure accuracy in various contexts.

This study contributes significantly to the existing literature on the use of point-of-care diagnostic devices, particularly by offering a comprehensive evaluation of the Scanbo device’s accuracy in measuring systolic and diastolic blood pressure and blood glucose levels. Unlike previous studies, which often focused on a single health parameter, this investigation provides a holistic view by simultaneously examining systolic blood pressure, diastolic blood pressure and blood glucose. The high level of accuracy and reliability demonstrated by the Scanbo device adds robust evidence supporting the integration of such technologies in community settings. This study not only confirms the efficacy of existing point-of-care devices, but also expands upon the potential clinical applications, paving the way for enhanced patient management and improved health outcomes.

This study boasts several strengths, including the use of standardised protocols for data collection (measurement of blood pressure and blood glucose) and the application of rigorous statistical methods to evaluate the accuracy of a new diagnostic device across multiple health parameters. Additionally, the use of Bland–Altman plots and ROC curve analysis provides a comprehensive assessment of agreement and diagnostic accuracy, which strengthens the validity of the conclusions drawn regarding the Scanbo device’s performance. While advanced molecular biomarkers such as DNA methylation patterns are being investigated for predictive diagnostics in cardiovascular diseases, tools like the Scanbo device provide immediate, non-invasive options for monitoring key health indicators in resource-limited settings.^18^

However, the study also has some limitations that must be considered. The sample size, while adequate for initial validations, is relatively small for establishing definitive conclusions, particularly in subgroups such as age and gender. This limits the ability to fully explore potential variabilities in device performance across different demographic groups. Moreover, the study’s cross-sectional design restricts the ability to assess the long-term reliability and accuracy of the device. The findings are based on single-time point measurements, which do not account for intra-individual variability over time. The use of convenience sampling to recruit participants may limit the generalisability of our findings, as this non-probabilistic sampling technique may not adequately represent the broader population, potentially introducing selection bias. Unmeasured factors could influence the outcomes, such as environmental conditions at the time of measurement or underlying health conditions not accounted for in the study design.

To maximise the utility of the Scanbo device, its integration into routine primary healthcare services, particularly at health and wellness centres (HWCs) under the Ayushman Bharat initiative, should be explored.^19^ As part of the Pradhan Mantri Jan Arogya Yojana (PM-JAY), the device could facilitate early detection and monitoring of hypertension and diabetes, aligning with the National Health Policy 2017 goal of reducing premature NCD-related mortality.^20^, ^21^ Its portability and user-friendly design make it ideal for deployment in remote areas, bridging diagnostic gaps. By offering cost-effective diagnostics, the device can reduce the financial burden on patients while enhancing the programme’s efficiency. Integration into a digital health ecosystem, with data feeding into state health systems, can further strengthen NCD management and policy planning. Such steps would enhance diagnostic capabilities, strengthen primary healthcare, and support India’s vision for universal health coverage.

## Conclusion

In conclusion, this study has demonstrated that the Bluetooth-enabled Scanbo device offers a high degree of accuracy and reliability in measuring blood pressure and blood glucose levels, with performance metrics closely aligning with those of established standard methods. These findings endorse the potential of the Scanbo device as a viable tool for routine monitoring and early detection of key health indicators in both clinical and community settings. Particularly noteworthy is the device’s capacity to integrate seamlessly into existing healthcare frameworks, thereby facilitating immediate decision-making and enhancing patient care. Future research should focus on refining these measurements and expanding the device’s capabilities to include a wider range of diagnostic parameters. By continuing to bridge the gap between point-of-care technology and traditional health monitoring methods, devices like Scanbo can significantly contribute to the global effort to manage and prevent chronic conditions effectively and efficiently.

## Declaration of generative AI and AI-assisted technologies in the writing process

During the preparation of this work the authors used ChatGPT to assist with drafting and language refinement. After using this tool, the authors reviewed and edited the content as needed and take full responsibility for the content of the publication**.**

## Ethics approval and consent to participate

This study was approved by the Institute Ethics Committee, Indian Institute of Public Health – Bengaluru Campus, Public Health Foundation of India (TRC – IEC No. IIPHHB/TRCIEC/212/2021, dated 04/01/2021) and conducted in accordance with the Declaration of Helsinki. Participants who were willing to participate in the study after obtaining written informed consent were recruited. Consent for the use of their data and images in research publications, with their identities appropriately masked to ensure confidentiality was also obtained from study participants.

## Funding

This research did not receive any specific grant from funding agencies in the public, commercial or not-for-profit sectors.

## Data availability statement

The data that support the findings of this study are available from the corresponding author upon reasonable request.

## CRediT authorship contribution statement

**Venkatesh Karthikeyan:** Writing – original draft, Visualization, Methodology, Conceptualization. **Sathyanarayana Tamysetty Narayan:** Supervision, Project administration, Methodology, Data curation, Conceptualization. **Chinnu Sara Varughese:** Writing – review & editing, Project administration, Data curation. **Shamshad Ahmad:** Writing – review & editing, Formal analysis. **Haripriya H:** Writing – original draft. **Mohit Bhardwaj:** Writing – review & editing, Formal analysis.

## Declaration of competing interest

The authors declare that they have no known competing financial interests or personal relationships that could have appeared to influence the work reported in this paper.
